# Transition From PCR-Ribotyping to Whole Genome Sequencing Based Typing of *Clostridioides difficile*

**DOI:** 10.3389/fcimb.2021.681518

**Published:** 2021-06-01

**Authors:** Helena M. B. Seth-Smith, Michael Biggel, Tim Roloff, Vladimira Hinic, Thomas Bodmer, Martin Risch, Carlo Casanova, Andreas Widmer, Rami Sommerstein, Jonas Marschall, Sarah Tschudin-Sutter, Adrian Egli

**Affiliations:** ^1^ Division of Clinical Bacteriology and Mycology, University Hospital Basel, Basel, Switzerland; ^2^ Applied Microbiology Research, Department Biomedicine, University of Basel, Basel, Switzerland; ^3^ Swiss Institute for Bioinformatics, Basel, Switzerland; ^4^ Institute for Food Safety and Hygiene, Vetsuisse Faculty, University of Zurich, Zurich, Switzerland; ^5^ Clinical Microbiology, Labormedizinisches Zentrum Dr Risch, Liebefeld, Switzerland; ^6^ Institute for Infectious Diseases, University of Bern, Bern, Switzerland; ^7^ Division of Infectious Diseases & Hospital Epidemiology, University Hospital Basel, University Basel, Basel, Switzerland; ^8^ Department of Infectious Diseases, Bern University Hospital and University of Bern, Bern, Switzerland; ^9^ Infectious Diseases, Hirslanden Central Switzerland, Lucerne, Switzerland

**Keywords:** *Clostridioides difficile*, ribotyping, whole genome sequencing, cgMLST, core genome, single nucleotide polymorphism, molecular epidemiology

## Abstract

*Clostridioides difficile* causes nosocomial outbreaks which can lead to severe and even life-threatening colitis. Rapid molecular diagnostic tests allow the identification of toxin-producing, potentially hypervirulent strains, which is critical for patient management and infection control. PCR-ribotyping has been used for decades as the reference standard to investigate transmission in suspected outbreaks. However, the introduction of whole genome sequencing (WGS) for molecular epidemiology provides a realistic alternative to PCR-ribotyping. In this transition phase it is crucial to understand the strengths and weaknesses of the two technologies, and to assess their correlation. We aimed to investigate ribotype prediction from WGS data, and options for analysis at different levels of analytical granularity. Ribotypes cannot be directly determined from short read Illumina sequence data as the rRNA operons including the ribotype-defining ISR fragments collapse in genome assemblies, and comparison with traditional PCR-ribotyping results becomes impossible. Ribotype extraction from long read Oxford nanopore data also requires optimization. We have compared WGS-based typing with PCR-ribotyping in nearly 300 clinical and environmental isolates from Switzerland, and in addition from the Enterobase database (n=1778). Our results show that while multi-locus sequence type (MLST) often correlates with a specific ribotype, the agreement is not complete, and for some ribotypes the resolution is insufficient. Using core genome MLST (cgMLST) analysis, there is an improved resolution and ribotypes can often be predicted within clusters, using cutoffs of 30-50 allele differences. The exceptions are ribotypes within known ribotype complexes such as RT078/RT106, where the genome differences in cgMLST do not reflect the ribotype segregation. We show that different ribotype clusters display different degrees of diversity, which could be important for the definition of ribotype cluster specific cutoffs. WGS-based analysis offers the ultimate resolution to the SNP level, enabling exploration of patient-to-patient transmission. PCR-ribotyping does not sufficiently discriminate to prove nosocomial transmission with certainty. We discuss the associated challenges and opportunities in a switch to WGS from conventional ribotyping for *C. difficile*.

## Introduction

*Clostridioides difficile* is an important pathogen, often associated with nosocomial outbreaks, but increasingly linked to community acquired infections (Durovic et al., 2018). While some patients can be asymptomatically colonized, *C. difficile* infection (CDI) is typically associated with antibiotic treated and immunosuppressed individuals (Ananthakrishnan, 2011). CDI can lead to severe colitis with sepsis and even fatal outcomes, yet the data on the relation between hypervirulent strains and adverse outcome remains conflicting (Wilson et al., 2010; Knight et al., 2015). Hypervirulent strains are those with: increased infectiousness relative to endemic strains; increased symptomatic disease rate relative to endemic strains; and an ability to outcompete endemic strains in the host’s gut (Yakob et al., 2015). Virulence may depend on several factors including the presence of toxins A and B and their repressor, and the binary toxin (encoded by *tcdA*, *tcdB, tcdC* and *cdtA*/*cdtB* respectively), the constitution of the *agr* locus (Knight et al., 2015), and trehalose metabolism (Collins et al., 2018). Hypervirulent *C. difficile* lineages carrying some or all of these factors have been defined (Stabler et al., 2006), and these factors may be used to guide both treatment and infection prevention and control recommendations (Widmer et al., 2017; Gerding et al., 2018; Tschudin-Sutter et al., 2018).

Typing is used to define lineages and trace epidemiological links, of which PCR-ribotyping is most commonly used for *C. difficile*. Hypervirulent lineages include RT027 and RT078 (Knetsch et al., 2011), among others. Other typing methods exist, including toxinotyping, serotyping, pulsed field gel electrophoresis (PFGE), Matrix assisted Laser Desorption Ionization Time of Flight (MALDI-TOF) mass spectrometry (Reil et al., 2011), and multi-locus sequence typing (MLST) (Griffiths et al., 2010). PCR-ribotyping analyses the intergenic spacer region (ISR) between the 16S and 23S rRNA genes (Gürtler, 1993). There are 11-12 copies of the rRNA operon in the genome (Sebaihia et al., 2006; Riedel et al., 2015; Kumar et al., 2018; Spinler et al., 2019), and there is variation among these copies in the ISR. PCR across the ISR (Bidet et al., 1999; Stubbs et al., 1999) generates fragments of different lengths, the sizes of which can be resolved by gel or capillary electrophoresis (Fawley et al., 2015). These band sizes can be compared against a database of isolates with known ribotypes either on-line (Indra et al., 2008) or in-house. The structure of, and diversity among, the ISRs has been investigated, showing the mosaic nature of the ISRs within and between isolate genomes, and suggesting intra- and inter-strain recombination as a source of variation (Sadeghifard et al., 2006; Indra et al., 2010). While providing higher discrimination than other typing techniques (Stubbs et al., 1999), this method is not fully portable between laboratories, is labor intensive, has a turnaround time of up to a week, and often requires in-house optimization. PCR-ribotyping has been a major typing technique for the past decades, but many clinical laboratories process to switch towards whole genome sequencing (WGS)-based typing (ECDC, 2019; Cho et al., 2020).

*C. difficile* is a genetically diverse species with a highly dynamic genome, with much of the variation driven by mobile elements and recombination (Sebaihia et al., 2006; He et al., 2010; Stabler et al., 2010). WGS gives access to the vast majority of the 4 Mb *C. difficile* genome, and the data provided are also comparable between centers. WGS also provides the highest discriminatory power for typing, which is critical for outbreak investigations and determination of patient-to-patient transmission. In most clinical laboratories using next generation sequencing for molecular epidemiology, short read sequencing has become standard. Using core genome MLST, such as that from Bletz et al. based on 2,270 genomic loci, putative transmissions can be identified, within a defined cluster threshold of ≤6 allele differences (Bletz et al., 2018). Single nucleotide polymorphism (SNP) analysis can refine this further for which a limit of two SNPs has been recommended to infer direct patient-to-patient transmission (Eyre et al., 2013).

While WGS provides excellent resolution of single copy regions of the genome, short read assemblies cannot reliably capture variation in repeat regions (Wick et al., 2017), such as the many copies of the ribosomal RNA operon and the repeated motifs within the ISRs (Sadeghifard et al., 2006; Indra et al., 2008). This means that ribotype-defining ISR fragment lengths cannot be determined from short read WGS (Bletz et al., 2018; Janezic and Rupnik, 2019; Goyal et al., 2020), although there is some agreement between ribotypes and MLST groupings (Griffiths et al., 2010; Dingle et al., 2011; Janezic and Rupnik, 2015; Frentrup et al., 2020). Using genome-wide association study (GWAS) methods, some genomic markers for ribotypes or ribotype groups have been identified (Goyal et al., 2020). WGS data can also be used to investigate the presence of virulence-associated factors, such as toxin-encoding genes (McLean et al., 2021) and antimicrobial resistance such as rifampicin resistance caused by mutations in *rpoB* (O’Connor et al., 2008; Isidro et al., 2018).

We aimed to investigate the correlation between MLST sequence type (ST), cgMLST, and PCR-ribotyping in clinical and environmental samples from Switzerland, against a background of global reference isolates.

## Methods

### Sample Collection

All samples were obtained from our routine laboratory at the University Hospital Basel, and originate from across Switzerland from 2015 to 2020. Additional recently published isolates, sent for PCR ribotyping and/or WGS typing from the Institute of Infectious Diseases at the University of Bern and the Labormedizinisches Zentrum Dr. Risch were included (Kuenzli et al., 2020). All are clinical isolates (n=238), environmental samples taken as swabs from patient rooms (n=39), or isolates from ring trials (n=17). All the patient samples are from separate or the same patient but with differing colony morphologies which showed double or sequential infection. Data have been anonymized, allowing analyses without ethical permission under the Swiss law and ethical regulations (Human Research Act) no ethical permission is required for a quality focused assessment of clinical samples.

### Diagnostics and Culture

Fecal specimens were screened for *C. difficile* glutamate dehydrogenase antigen by C.DIFF CHEK-60 immunoassay (TechLab). Screen-positive stool samples were further tested for toxin production by Xpert^®^ *C. difficile* (Cepheid). If the following three targets are detected by Xpert^®^: toxin B (*tcdB*), binary toxin (*cdtA*), and a *tcdC* deletion at nucleotide 117, a presumptive identification of the 027 epidemic strain is reported. A presumptive identification of 078 epidemic strain may be reported if toxin B (*tcdB*) and binary toxin (*cdtA*), but not the *tcdC* deletion, are detected. *C. difficile* culture was used prior to confirmation of putative epidemic strains by PCR-ribotyping. Shortly, stool specimens were plated on selective cycloserine-cefoxitin-fructose agar plates (CLO agar; bioMérieux) and incubated in an anaerobic chamber for 48 h according to standard laboratory methods. Colonies were identified as *C. difficile* by MALDI-TOF MS (Bruker Daltonics).

### PCR-Ribotyping

PCR-ribotyping was performed under accreditation (ISO/IEC 17025) as previously described (Widmer et al., 2017) using high-resolution capillary gel-based electrophoresis (Indra et al., 2008) and primers as described (Stubbs et al., 1999). Capillary electrophoresis was conducted using the automated sequencer used the ABI-3500 Genetic Analyzer (Applied Biosystems [Life Technologies], Foster City, CA). Fragments were analyzed using GeneMapper v 5.0 (Applied Biosystems) and ribotype patterns compared in Bionumerics v 7.6.2 (Applied Maths, Sint-Martens-Latem, Belgium) software. Fragment profiles were compared with those generated using the standard set of the ECDC Brazier strain collection, which was obtained from the European *Clostridium difficile* infection study network (ECDIS-NET). Isolates for which no matching ribotype was found in our database were sent to the laboratory of Prof. E.J. Kuijper, Leiden University Medical Centre (LUMC), Leiden, the Netherlands, and when necessary onward to the laboratory of Prof. M.H. Wilcox, School of Medicine, University of Leeds, Leeds, United Kingdom for comparison with larger European databases.

### Whole Genome Sequencing

DNA was extracted using EZ1 Advanced XL (Qiagen, Hilden, Germany). DNA was sequenced on the Illumina MiSeq (300 bp paired end reads) or NextSeq (150 bp paired end reads) platforms following NexteraXT or Nextera Flex library creation. All were sequenced to over mean 42x coverage with NexteraXT and over 30x with Nextera Flex. Six isolates underwent long read Oxford Nanopore Technologies (ONT) sequencing on a GridION platform following Barcode ligation library protocol. All read data are available from the European Nucleotide Archive (https://www.ebi.ac.uk/ena/) under project PRJEB43401.

### Whole Genome Analysis and GWAS

Illumina read data was assembled using Unicycler v0.3.0b (Wick et al., 2017). ONT data was filtered using filtlong (https://github.com/rrwick/Filtlong) to remove reads under 1kb and retain 50-95% of the data, leaving over 250x coverage. Quality control data shown in **Figure S1** shows that read lengths were sufficient to straddle rRNA operons (>7kb). Reads were assembled in parallel using either canu (Koren et al., 2017) or flye (https://github.com/fenderglass/Flye), followed by polishing using filtered Illumina reads (Chen et al., 2018) and pilon (Walker et al., 2014) for ten iterations. Hybrid assembly was also performed on filtered reads using Unicycler 0.4.8, all using default settings. Mean coverage and assembly data for canu, flye, and unicycler are given in **Table S1**.

MLST and core genome MLST (cgMLST) were analyzed within Ridom SeqSphere+ v6.0.2 (Jünemann et al., 2013) using Unicycler Illumina only assemblies. The standard Ridom Seqsphere+ quality cutoff requires >90% of alleles to be found in the assemblies (up to 227 missing). which was found to provide fewer missing alleles than Velvet in Ridom (**Figure S2**) and the defined schemes (Griffiths et al., 2010; Bletz et al., 2018). Genome data with ribotype metadata was downloaded from Enterobase on 05.02.2019 (n=2456) (Frentrup et al., 2020; Zhou et al., 2020). GWAS for RT078 versus RT126 were carried out using DGBWAS 0.5.4 (Jaillard et al., 2018) with default parameters. DBGWAS is a kmer based approach to detect genetic variants underlying a phenotype, covering SNPs, indels, and gene presence/absence. Q-values reported by DBGWAS correspond to Benjamini-Hochberg transformed p-values controlling for false discovery rate.

Data from Nextera Flex libraries provided better assemblies compared to those from NexteraXT libraries (**Figure S2**), due to the greater evenness of coverage provided (Seth-Smith et al., 2019). Nine genomes were excluded due to poor quality.

In silico PCR-ribotyping was performed on all long read assemblies (six isolates and five assembly methods) using the Bidet primers 5’-GTGCGGCTGGATCACCTCCT-3’ (16S primer) and 5’-CCCTGCACCCTTAATAACTTGACC-3’ (23S primer) (Bidet et al., 1999) and in_silico_pcr (https://github.com/egonozer/in_silico_pcr). Resulting predicted band sizes were compared against those exported from the Bionumerics software for the given ribotypes. To investigate the assembly level of rRNA operons, contigs carrying 16S or 23S rRNA genes were detected using BLAST (Altschul et al., 1990) and respective contig lengths determined using samtools (Li et al., 2009).

### Simpson’s Diversity Calculations

Simpson’s index of diversity between ribotypes and MLST sequence types was calculated for 2073 isolates at http://www.comparingpartitions.info/ (Carriço et al., 2006).

## Results and Discussion

### Diversity of *C. difficile* isolates in Our Swiss Collection

**Figure 1:**
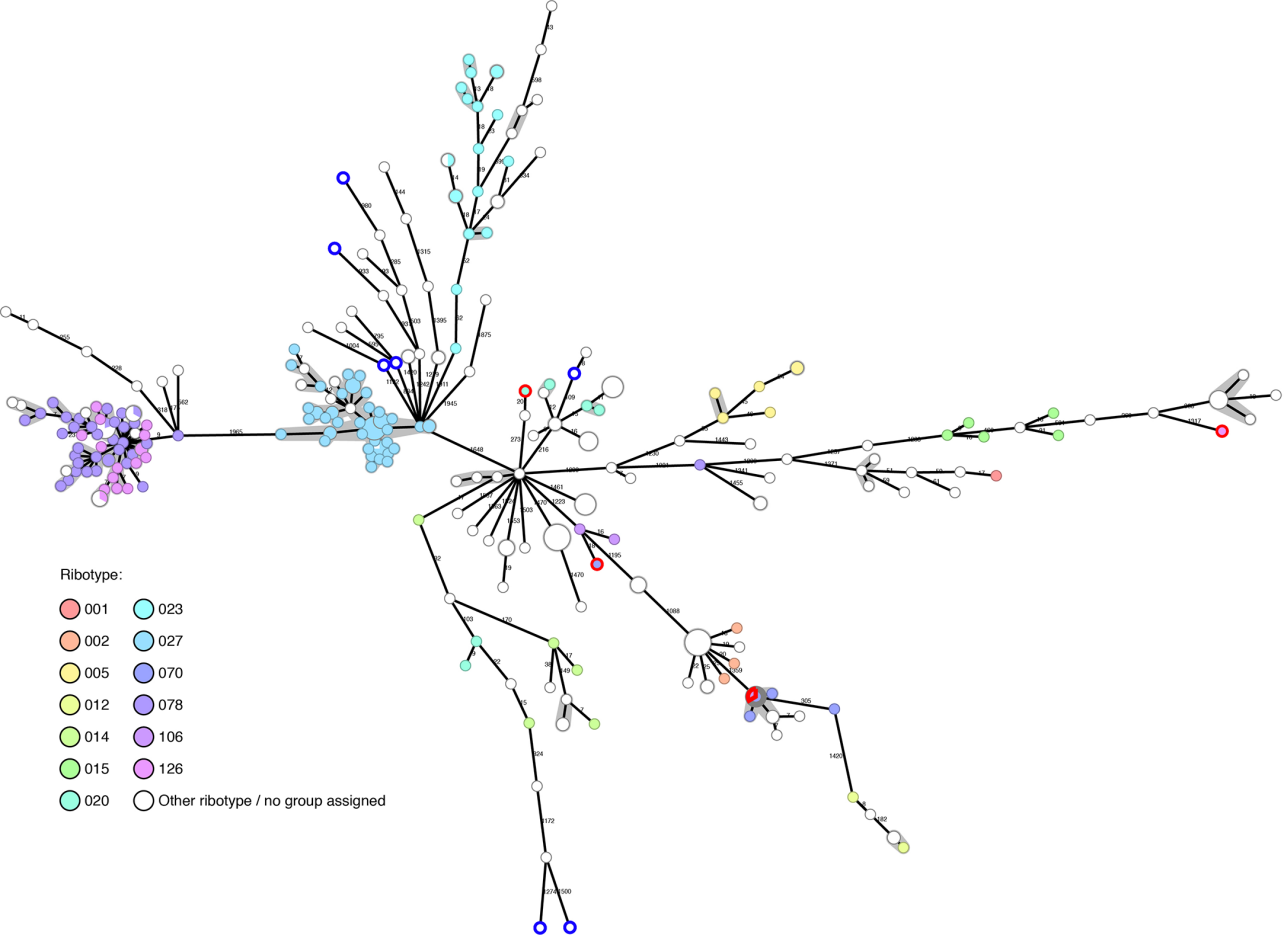
Basel sequenced isolates (n=294): cgMLST colored by ribotype. Nodes are colored by ribotype and size corresponds to the number of isolates at that node. Distances are shown on edges, other than those ≤6, which are shown with the grey shadowing. The seven genomes with blue outlines are those that could not be PCR-ribotyped. The four genomes with red outlines were later found to have questionable ribotype assignments.

Of our collection of 294 in-house sequenced *C. difficile* isolates, PCR-ribotyping results are available for 177, showing that they belong to 36 different ribotypes. A further seven have been PCR-ribotyped, but no matching ribotype could be determined using either our own ribotype database or through external PCR-ribotyping at different centers (University of Leiden and University of Leeds). The ribotypes detected are: RT027 (n=44); RT078 (n=33); RT126 (n=16); RT023 (n=13); RT005 (n=6); RT020 (n=6); RT070 (n=6); RT014 (n=5); RT015 (n=5); RT207 (n=4); RT002 (n=3); RT033 (n=3); RT106 (n=3); RT012 (n=2); RT050 (n=2); RT057 (n=2); RT111 (n=2); RT122 (n=2); RT251 (n=2); RT267 (n=2); and one genome each belonging to RT001, RT009, RT010, RT013, RT016, RT029, RT034, RT036, RT039, RT045, RT087, RT131, RT150, RT153, RT163, RT250. The diversity of the *C. difficile* genomes was calculated by cgMLST and is represented as a minimum spanning tree (MST) in **Figure 1**. WGS typing was requested mainly for outbreak investigations, and not as random sample of all *C. difficile* patient isolates: nevertheless, we observed a wide diversity of isolates in our Basel collection over the past five years.

The genomes tend to cluster in ribotype groups. RT027 (n=44) includes isolates from a previously described outbreak (Kuenzli et al., 2020) and shows a maximum diversity between isolates of 12 allelic differences. Within the RT078/126 cluster (n=49), the maximum diversity between two genomes is nine allelic differences. Further ribotypes cluster, but are more disperse, such as RT023 (n=13, max allele difference=52) and RT015 (n=5, max allele difference=464).

The gain in resolution given by cgMLST, in comparison to ribotype alone, helps to rule out transmission in many cases where PCR-ribotyping would be potentially misleading (**Figure 1**).

### PCR-Ribotyping and MLST Comparison in the Background of Global Reference Isolates

In order to put our data in the context of a global collection, we downloaded all genomes from the Enterobase database with allocated ribotypes and imported the genomes with metadata into Ridom Seqsphere+. After down sampling the dataset to remove many genomes identical by cgMLST, and quality controlling for >90% of alleles found, n=1778 genomes remained. Combined with our collection of genomes, these represent 141 ribotypes (**Table S2**). While these genomes from isolates with known ribotypes are indeed diverse, biases in the dataset remain, with many genomes from Germany (n=608), United Kingdom (n=368), Spain (n=225) United States (n=64), and Canada (n=53), as well as the n=294 from Switzerland: further countries were each represented by fewer than 50 genomes.

Our collected genomes from Switzerland are dispersed within the global genomes (**Figure S3**), suggesting that many strains are seeded into the area from abroad, rather than there being only Switzerland-specific strains circulating. Seeding can come from many sources, and may be arriving at the hospital from the community (Durovic et al., 2018).

MLST was calculated from genome sequences, showing that the collection has 118 STs; eight samples have novel or unknown STs. PCR-ribotyping shows a slightly higher degree of discrimination than MLST, as the former has a Simpson’s index of diversity of 0.901 (CI 0.894-0.908), and MLST of 0.881 (CI 0.873-0.889). Complex type as defined by cgMLST has a Simpson’s index of diversity of 0.911 (0.900-0.921), showing its greater ability to discriminate (p=0.130).

**Figure 2:**
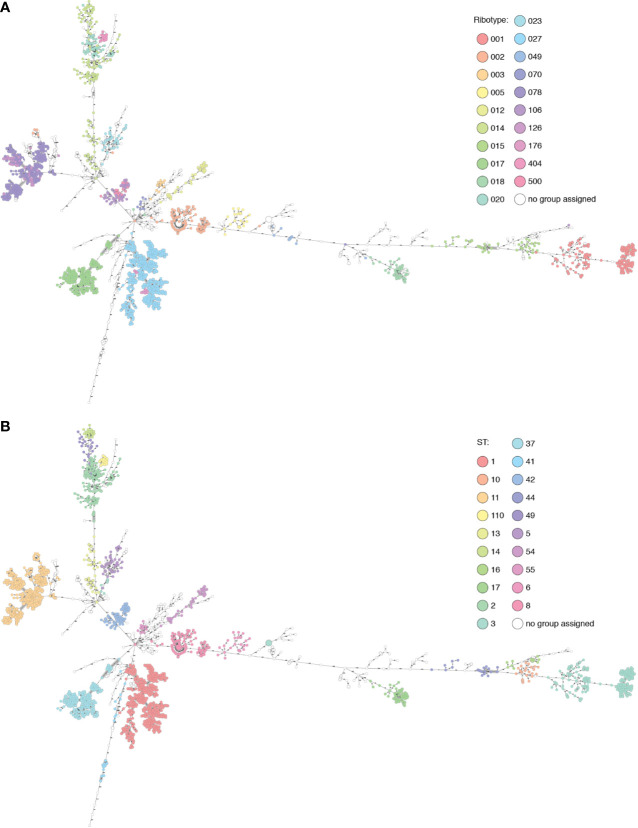
The Basel collection and selected Enterobase genomes displayed in Ridom Seqsphere+ (n=2094). **(A)** Nodes colored by ribotype. **(B)** Nodes colored by ST. Many overlaps of ribotype and ST can be seen, also cases where multiple ribotypes are found within the same cluster (RT106/500) described by one ST (42), or where diverse ribotypes (015) are split into multiple clusters corresponding to several STs (10, 44 and 160).

As with ribotypes, sequence types (STs) also cluster in the MST, and in many cases the ribotype clusters largely match ST clusters (**Figure 2**, **Table S2**). In several cases, from our dataset and also from Enterobase data, the genome was found not to cluster with others from that ribotype: these may be mis-ribotyped and should be treated with caution.

RT017 (n=191) shows a maximum diversity of 22 alleles between genomes within the cluster, separated from other ribotypes by over 100 alleles, and has a unique correspondence with ST37. RT005 (n=20) is uniquely linked to ST6, with up to 41 alleles between genomes and over 1000 to other ribotypes.

RT015 (n=40) is more diverse, with up to 449 alleles between isolates of this ribotype, separated from others by 585 or more alleles. This diversity is reflected in the MLST clusters within this ribotype: ST10, ST44, and ST160. In this case, ST provides higher resolution than PCR-ribotyping. In a counter-example, all RT001 (n=146) genomes belong to ST3, in an ST3 cluster with genomes from RT044, RT072, RT077, RT241, RT456, and RT559, some of which are only two alleles from RT001 genomes (data not shown). ST3 is also found polyphyletically in distant clusters in the MST, correlating with RT009/RT262 and RT305, so in this case MLST distinction alone does not provide sufficient resolution.

The RT027 (n=426) ST1 cluster contains also RT176 (n=12). Most genomes differ by under 13 alleles, although there are some longer branches of 57, 117, and 184 allelic differences in the cluster. Within the cluster is a subcluster of RT017 ST417 isolates (n=7), with 10 allelic differences to the closest ST1 genome. One branch of 147 alleles leads to an RT027 isolate with ST371. Similarly, RT106 (n=51) is intermingled with RT500 (n=20) in an ST42 cluster, with supposedly different ribotypes having identical core genomes in three cases. All genomes cluster with under 18 allele differences except in one case with a branch length of 68 (RT106, ST28). This cluster is separated by over 1000 allele differences from other genomes.

Within the known ribotype complexes, including RT078/126 and RT014/020/207, genomes cluster together. The latter group is described in higher resolution by the STs 2, 14 and 49 with 144-150 alleles between each. The diversity within these ST clusters is generally low (<30 alleles) with the exception of two longer branches within RT014/ST2 of 148 and 232 alleles. RT404 corresponds to ST110 and is also linked to this cluster, separated by 104 alleles. This cluster alone shows how specific cutoffs between clusters cannot be used to fully define either ribotypes or STs.

MLST and PCR-ribotyping schemes are based on different genomic features, so full concordance of clusters would not be expected. It can be argued that MLST should be more representative of the underlying genomic ancestry and phylogeny of the isolates, especially as the mechanisms behind the ISR patterns are not fully understood (Indra et al., 2010). Unique correlations between ribotype and ST are not the rule; when these data are superimposed with cgMLST resolution, we can see that ribotype distinctions do not always infer ancestry, confirming what has been previously noted (Frentrup et al., 2020). In the cases of the RT001 cluster, RT014/020/207, RT027/176, RT078/126, and RT106/500, it is arguable whether PCR-ribotyping provides useful resolution, or whether these discriminations are largely arbitrary and could potentially lead to missed epidemiological connections. ST distinctions correlate better with the phylogeny, but in a recombining bacterium such as *C. difficile*, MLST also provides anomalies such as in the case of ST3.

Neither ST nor cgMLST can thoroughly distinguish between RT078 and RT126. This distinction may be of clinical and/or epidemiological importance, given that the association with more severe disease manifestation has been mainly reported for RT078 so far and that RT078 may have a higher incidence in healthcare settings (Goorhuis et al., 2008; Bauer et al., 2011; Freeman et al., 2018). We chose to explore whether there is genomic evidence to support this distinction. In a GWAS approach for RT078 (n = 372) versus RT126 (n = 61), we did not identify any SNPs, indels, or genes that defined either ribotype. Although multiple genes were strongly associated with RT126 (such as group II intron reverse transcriptase (accession SJP52541.1), frequency 92% vs 8% in RT078, minimum q-value = 2.72 × 10^-10^), and found within a phylogenetic cluster dominated by RT126 isolates, they did not serve as discriminatory markers or suggest a role in virulence (data not shown). Previously reported RT078-associated markers (Goyal et al., 2020) were also found in RT126.

What is clear from these data is that cgMLST, based on thousands of loci rather than a mere handful, provides higher resolution and more interpretable information to inform epidemiological investigations, than either PCR-ribotyping or MLST.

### Predicting Ribotype and Determining cgMLST Cutoffs Between Ribotype Groups

**Figure 3:**
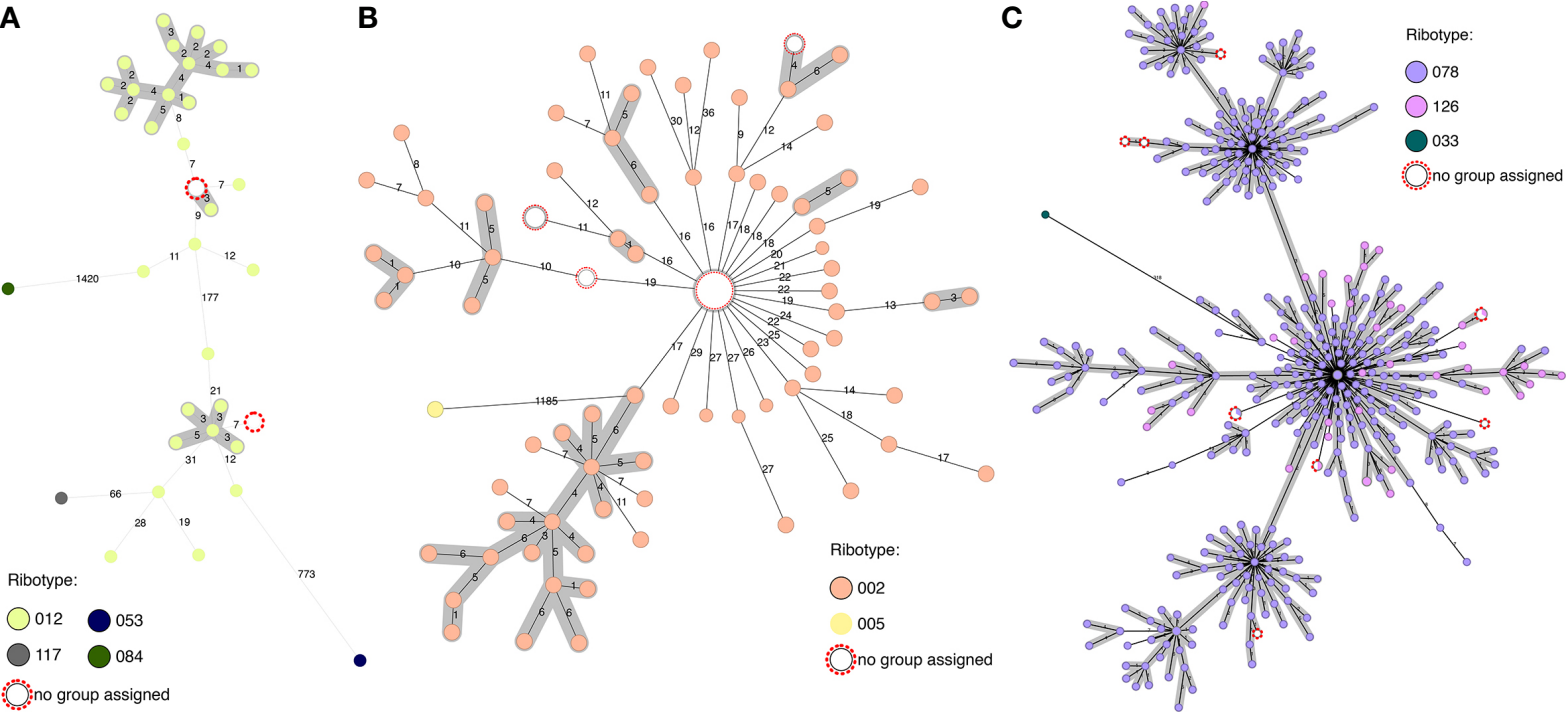
Predicting ribotype from cgMLST through comparison to genomes with known ribotype. **(A)** RT012 cluster, with two samples with unknown ribotype (max 7 alleles from other isolates) and closest related ribotypes. **(B)** RT002 cluster, with samples with unknown ribotype (max 16 alleles from other isolates) and closest related ribotype. **(C)** RT078/126 cluster, with samples with unknown ribotype and closest related ribotype (max 9 alleles from other isolates).

Investigating within clusters, we can see how in some cases it is possible to determine ribotypes from the cgMLST data (**Figure 3**). In clusters of RT002 and RT012 we can be fairly certain of the ribotype identity of the non-PCR-ribotyped samples, as they are under 20 alleles from other samples known to have that ribotype. In the RT078/126 cluster, this is not the case as ribotypes are intermingled in the cluster, such that samples can only be assigned to this ribotype complex. In terms of epidemiological tracking, however, it is the identity between the genomes, and not the ribotype which provides the most valuable information.

Of the 110 isolates with genomes in our collection for which no PCR-ribotyping has been performed, ribotypes or ribotype clusters can be predicted from the combined database for 78 samples within 50 alleles, of which 74 are within 30 alleles of at least three genomes of known ribotype. The 32 samples for which ribotypes cannot be assigned are either >300 alleles from samples with a known ribotype, >50 alleles from only a single sample with a known ribotype, or similarly related to several samples with different ribotypes. In these cases, ribotype cannot be assigned with certainty.

To facilitate the assignment of samples to ribotypes within such a framework, it has been suggested that allele number cutoffs could be determined using distance matrices (**Figure S4**) (Frentrup et al., 2020). Frentrup et al. propose that ribotypes can in many cases be assigned as being within 150 alleles of genomes with known ribotypes (hierarchical clustering HC150 clusters), and epidemics within HC10 (10 alleles). However, as we saw above, different ribotypes have different cluster densities (ie different allele distances within the cluster) and different allele distance cutoffs to other ribotypes. There may also be different dynamics occurring within varied ribotypes, as some such as RT027 recently expanded very rapidly (He et al., 2013), and this and RT078 are the most commonly observed ribotypes in our collection. Interpretation of cluster differences in these ribotypes may be different to interpretation in more diverse ribotypes such as RT014 and RT020. The impact of recombination should also be taken into account. With all these considerations, tailored analysis by a bioinformatician is preferable to having defined cutoffs.

We used the Ridom SeqSphere+ software and schema, which was previously found to have a lower discriminatory power than the 2,556 gene schema implemented within Enterobase (Frentrup et al., 2020). Many clinical routine laboratories run Ridom SeqSphere+, so this comparison is relevant to many. The discriminatory power is linked to the NGS library method and assembly algorithm, as the more alleles which can be used within the scheme, the higher the accuracy (Frentrup et al., 2020).

### Defining Ribotype From Long Read Sequencing Data

In assemblies of short read Illumina data, rRNA genes collapse into single copies. We analyzed 114 randomly selected Illumina-only assemblies from various ribotypes among our in-house dataset. In all, the 16S and 23S rRNA genes were either not assembled or found on two distinct single-gene contigs, thus precluding an in-silico analysis of the ISR. Because ISRs often consist of repetitive modules (Sadeghifard et al., 2006), it is also not possible to determine ribotypes using read mapping based approaches for short-read data.

To determine the ease of extracting ribotype from long read data, we sequenced six isolates with known RT by ONT: two RT078; two RT126; one RT020; and one RT070. PCR-ribotyping band sizes were extracted from Bionumerics and compared to the calculated in silico PCR sizes from several different assemblies of the data (**Table S3**).

We find that many of the correct band sizes are predicted, but that no single assembler appears to be optimal across all genomes tested. For two genomes (306515 and 301392), all assemblers produced equally good results. Hybrid assemblies, using Illumina reads to polish the ONT data, improved ISR calling in isolate genome 360432, 302561 and 302200, but ONT data alone gives the best results for genome 359991. The band distinguishing RT078 from RT126 (446bp from Bionumerics) was not consistently accurately called, which would lead to inaccurate ribotype calling within this complex.

Three well assembled genomes from RT078 and RT126 (unicycler hybrid assemblies of 306515, 301392 and 302200), for which the ISR assemblies match the PCR fragment pattern (**Table S3**), were compared, to investigate the source of the additional band in RT078 that is not found in RT126. In these genomes, 11 of the 12 ISRs were found to be identical. A deletion of a 42 bp direct repeat module was however detected in the ISR of *rrnA* (flanked by *sigB*, Gürtler, 1993) in the two RT126 genomes, explaining their distinct PCR ribotype patterns. Repeated loss and/or duplication of this repeat may underlie the polyphyletic emergence of RT126 from within RT078 (**Figure 2**, Knight et al., 2019; Frentrup et al., 2020).

This is a small and preliminary dataset, but illustrates the challenges associated with such analysis: further studies are clearly needed.

## Conclusions

NGS has become the new gold standard of bacterial typing (Dylus et al., 2020) and transition from other typing technologies such as PCR-ribotyping is ongoing. Currently, PCR-ribotyping is recommended for characterization of *C. difficile* isolates for CDI surveillance purposes, and is the currently favored approach by the European Centre for Disease Prevention and Control (ECDC) (Krutova et al., 2018). Thus switching from PCR-ribotyping to WGS may hamper participation in multinational surveillance schemes and impede comparability of current strain distribution between institutions and countries. Such schemes are important to understand the current epidemiology of *C. difficile* on a broader scale. As many centers are likely to switch to WGS in the near future, and abandon other typing methodologies, a coordinated approach would be favorable. As such a transition phase may take time, important outbreak clones, as well as novel introductions of animal-related *C. difficile* strains into the human population may be missed due to the lacking or hampered comparability of the results obtained by different typing approaches. Therefore, a two-step approach, consisting of first, PCR-ribotyping and second, performing WGS to investigate suspected outbreak-clusters, as has recently been suggested (Krutova et al., 2019), may be reasonable in such a transition phase.

WGS data allow analysis at different levels of resolution from MLST, cgMLST, to SNP differences. We show that the cgMLST schemes from Enterobase (Frentrup et al., 2020) and Ridom SeqSphere+ (Jünemann et al., 2013; Bletz et al., 2018) give very similar, high resolution results, and even more rapid tools for genome clustering are being developed (Eyre et al., 2019). However, cgMLST does not provide the ultimate resolution, and SNP level analysis is preferable for the accurate analysis of suspected transmissions or outbreaks. An outbreak of RT027 in Switzerland showed that genomes from one center clustered within three allele differences, well within the ≤6 allele cluster limit (Kuenzli et al., 2020). A SNP tree of the outbreak associated isolates showed more than 100 SNP differences between some of the isolates, and in this case the higher resolution was useful in characterizing the outbreak (Kuenzli et al., 2020).

In many cases, RT can be predicted from genome assemblies *via* cgMLST, but sequenced reference genomes are required for comparison. Rapid, long read technology may be used for ribotype prediction in the future, but requires further bioinformatic method optimization. Given the increasing number of publicly available genomes, GWAS analysis may be able to identify ribotype-predictive markers from short-read assemblies. Several highly selective polymorphisms have been identified for five important ribotypes (Goyal et al., 2020). Yet, the genomic and clinical distinction between RT078 and RT126 remains unclear. Further studies are necessary to expand the collection of markers and to validate their robustness.

NGS data can also be probed for virulence determinants (toxin-encoding) and antimicrobial resistance determinants, providing useful clinical information regarding the manifestations of infection with an evolving pathogen. This analysis could be carried out using a custom scheme within Ridom Seqsphere+, which would otherwise require additional diagnostic tests. While data provided from NGS on typing and clusters are very valuable, when it is combined with clinico-epidemiological data, outbreaks can be better interpreted, and more patient- and healthcare-relevant conclusions can be drawn.

Rapid diagnostic tests for the presence of toxins (for example GeneXpert *C. difficile* BT) can provide much of the information required to assign patients to isolation precautions. PCR-ribotyping continues to be required for international comparison. Higher resolution is often desirable, particularly in cases of suspected nosocomial transmission. WGS, particularly as speed increases and costs decrease, is set to become the *C. difficile* typing method of choice.

## Supplementary References

(Team, R; Team, RC; Wickham, 2016; De Coster et al., 2018)

## Data Availability Statement

The datasets presented in this study can be found in online repositories. The names of the repository/repositories and accession number(s) can be found below: https://www.ebi.ac.uk/ena, PRJEB43401.

## Author Contributions

The project was planned by AE and HS-S. Samples were provided by TB, MR, CC, RS, AW, JM, and ST-S. Microbiological analysis was carried out by VH. Analysis was carried out by HS-S and MB. Whole genome sequencing was supervised by TR. The manuscript was drafted by HS-S, MB and AE. All authors contributed to the article and approved the submitted version.

## Conflict of Interest

The authors declare that the research was conducted in the absence of any commercial or financial relationships that could be construed as a potential conflict of interest.
