# Supplementary figures and images for: Transition From PCR-Ribotyping to Whole Genome Sequencing Based Typing of Clostridioides difficile
Source: Front Cell Infect Microbiol. 2021 Jun 1;11:681518. doi: 10.3389/fcimb.2021.681518 (PMC8204696; doi:10.3389/fcimb.2021.681518)

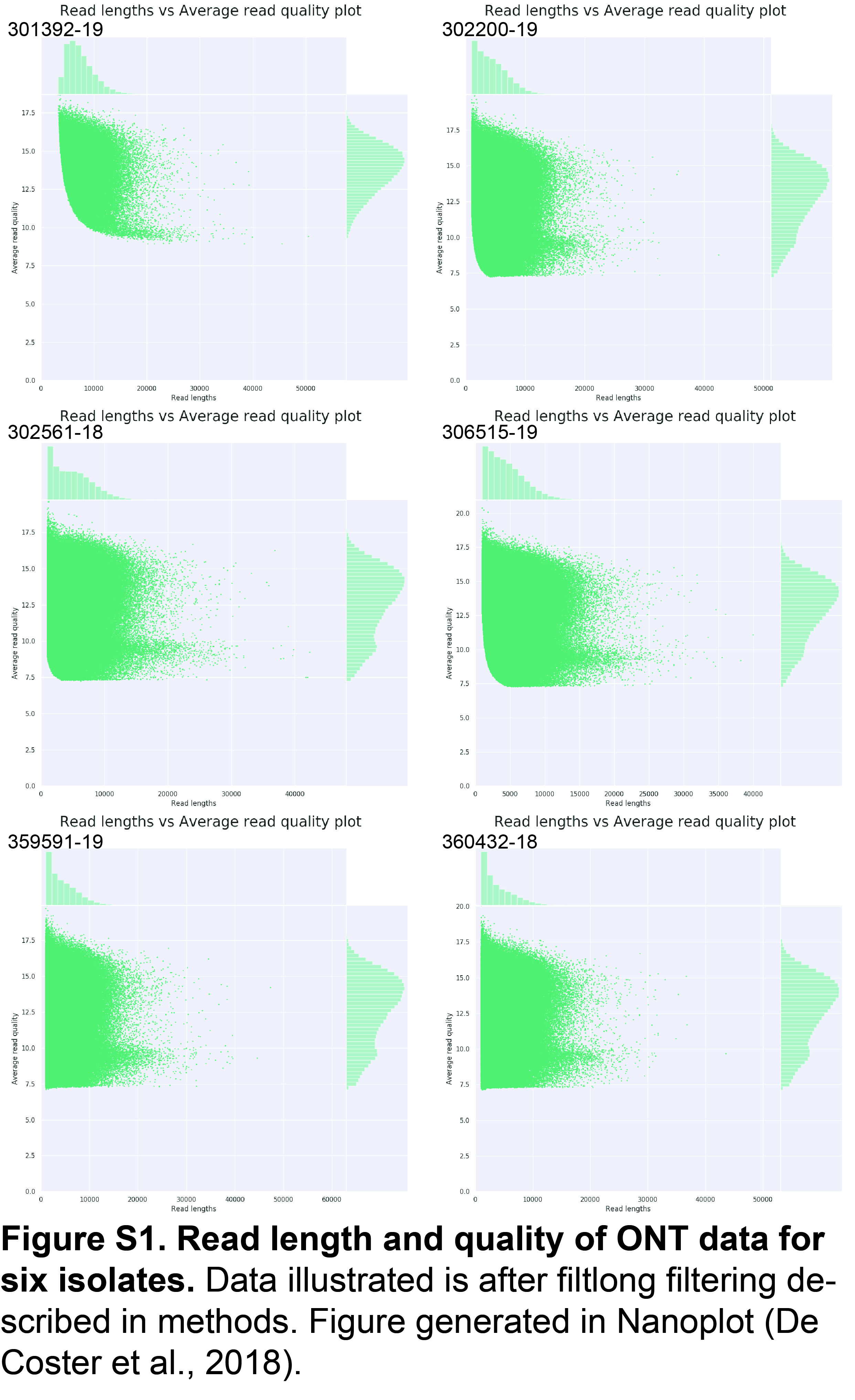

Supplement: Supplementary file 1 [file Image_1.jpeg]

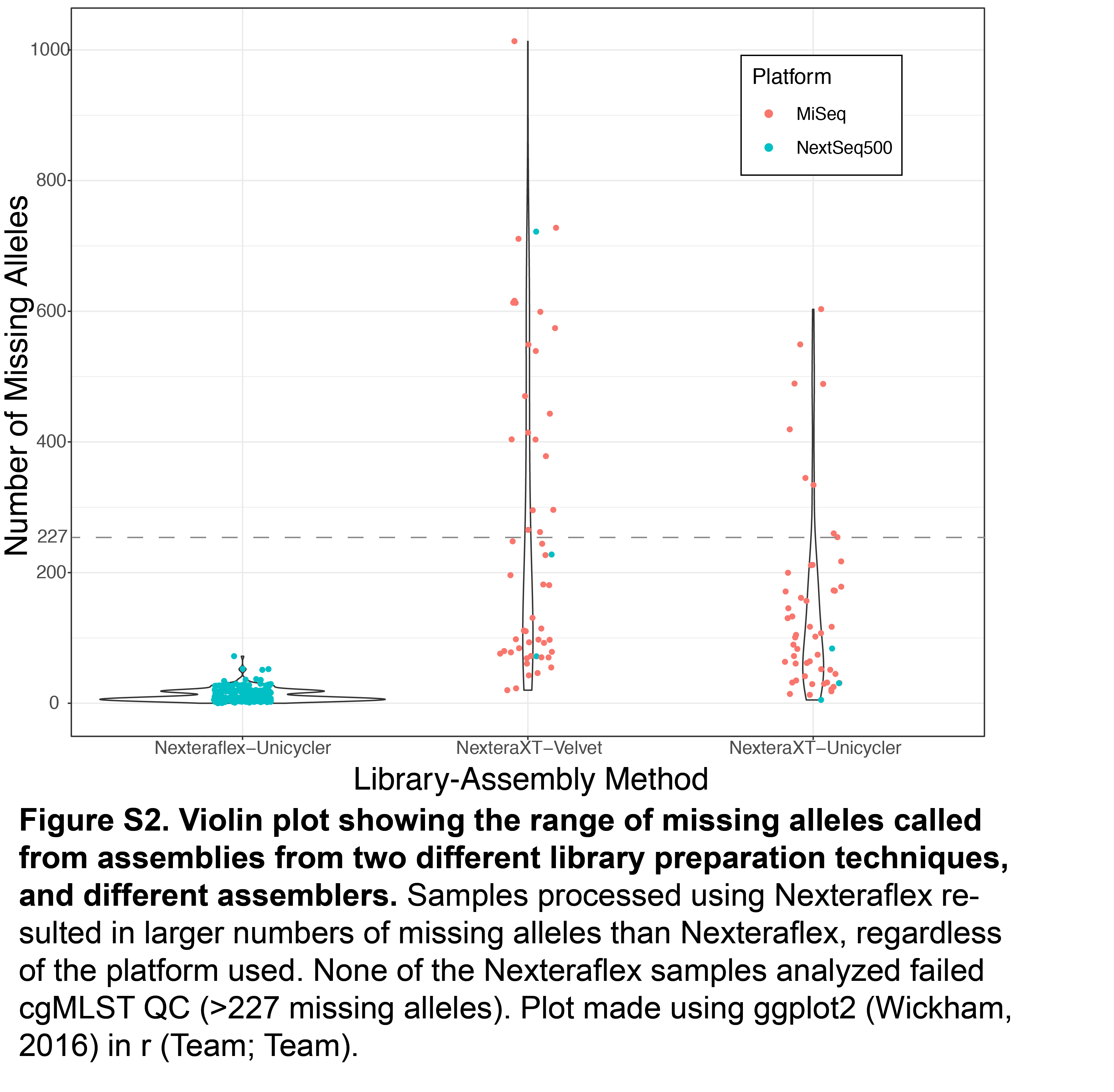

Supplement: Supplementary file 2 [file Image_2.jpeg]

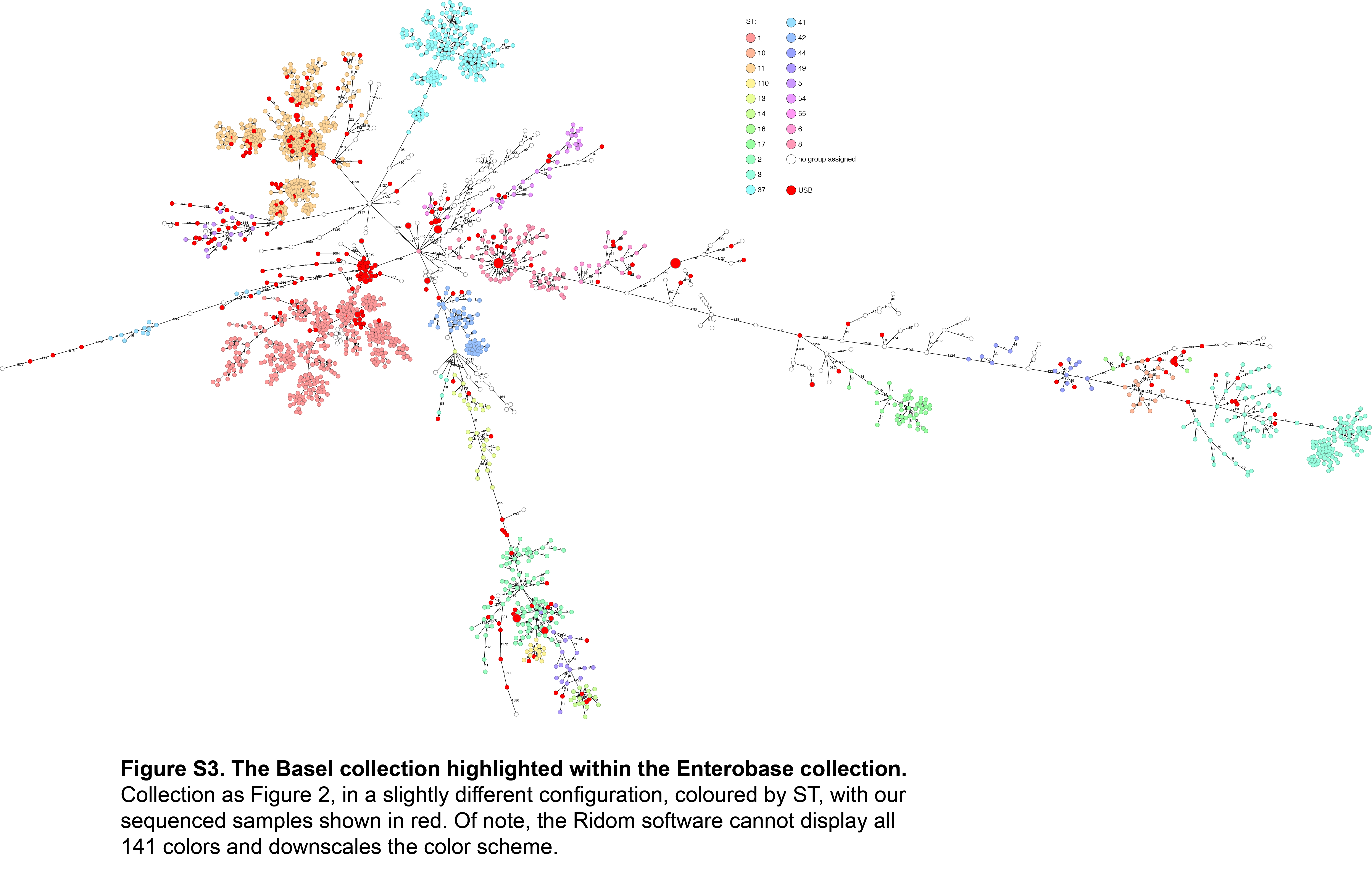

Supplement: Supplementary file 3 [file Image_3.jpeg]

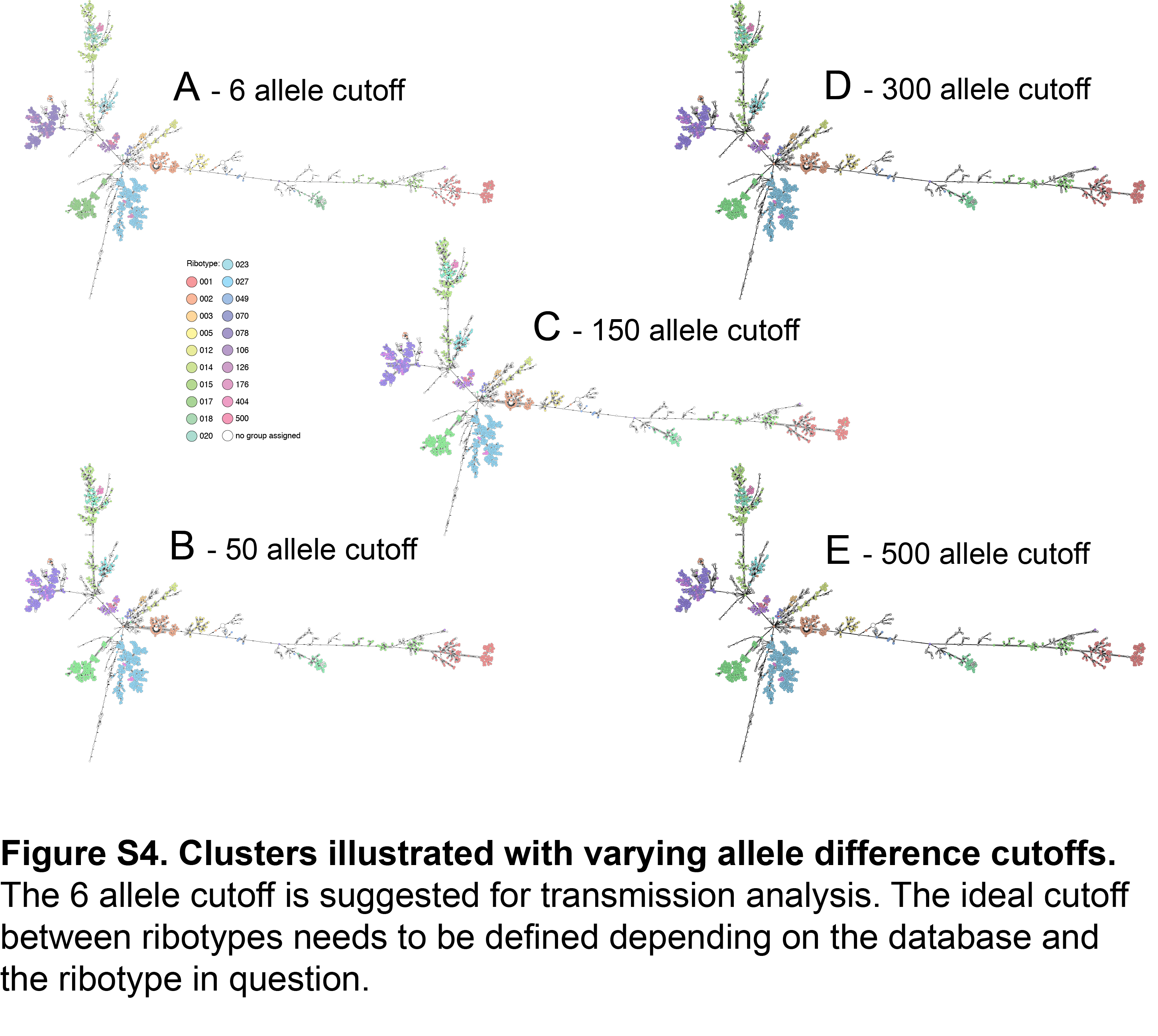

Supplement: Supplementary file 4 [file Image_4.jpeg]
