# Supplementary material for: Transition From PCR-Ribotyping to Whole Genome Sequencing Based Typing of Clostridioides difficile
Source: Front Cell Infect Microbiol. 2021 Jun 1;11:681518. doi: 10.3389/fcimb.2021.681518 (PMC8204696; doi:10.3389/fcimb.2021.681518)
Supplement: Supplementary file 5 [file Table_1.docx]

Supplementary Tables

**Table S1. Genome coverage and assembly parameters for six isolates.** Long read assemblies were performed using: canu, with and without pilon polishing with Illumina reads; unicycler hybrid assembly, and Flye with and without pilon polishing with Illumina reads.

| Isolate |  | 301392-19 | 302200-19 | 302561-18 | 306515-19 | 359591-19 | 360432-18 |
| --- | --- | --- | --- | --- | --- | --- | --- |
|  | RT | 126 | 126 | 070 | 078 | 078 | 020 |
|  | Illumina mean coverage | 157 | 155 | 120 | 66 | 78 | 111 |
|  | ONT mean coverage | 488 downsampled to 259 | 461 | 290 | 592 | 347 | 409 |
| Canu | Total assembly length | 4196813 | 4144492 | 4270041 | 4086592 | 4402348 | 4209774 |
|  | #contigs | 9 | 9 | 10 | 18 | 21 | 10 |
|  | Longest contig | 3979949 | 2064610 | 2964413 | 2101959 | 4107764 | 4120648 |
| Canu+Pilon10 | Total assembly length | 4244546 | 4182509 | 4537811 | 4114479 | 4818370 | 4396742 |
|  | #contigs | 9 | 9 | 10 | 18 | 21 | 10 |
|  | Longest contig | 4025651 | 2092133 | 3080601 | 2114676 | 4479843 | 4299023 |
| Unicycler | Total assembly length | 4224888 | 4103072 | 4104252 | 4039798 | 3918318 | 4260907 |
|  | #contigs | 38 | 11 | 19 | 7 | 15 | 42 |
|  | Longest contig | 4129293 | 2103368 | 3543713 | 3987269 | 1419360 | 1661562 |
| Flye+3polishiterations | ONT cov | 254 | 457 | 282 | 603 | 336 | 402 |
|  | Total assembly length | 4114992 | 4043528 | 4152891 | 3954799 | 4140890 | 4116845 |
|  | #contigs | 2 | 2 | 3 | 2 | 5 | 1 |
|  | Longest contig | 2901317 | 4033329 | 3341926 | 3931922 | 2277655 | 4116845 |
| Flye+Pilon10 | Total assembly length | 4151189 | 4069060 | 4351995 | 3968000 | 4403841 | 4254266 |
|  | #contigs | 2 | 2 | 3 | 2 | 5 | 1 |
|  | Longest contig | 2919876 | 4058851 | 3477625 | 3945098 | 2486985 | 4254266 |

**Table S2. Samples sequenced in Basel, used in this study (n=294).** NP: not performed.

| **Table 1. Samples sequenced in Basel, used in this study (n=294).** NP: not performed. | | | | | | | |
| --- | --- | --- | --- | --- | --- | --- | --- |
| Sample ID | Collection Date | ST | Ribotype | #Missing cgMLST alleles | Perc. Good cgMLST alleles | Source | Location of submitting laboratory (Canton) |
| 103V-ZC1 | 02.06.2020 | 3 | NP | 9 | 99.6 | Environmental | Basel City |
| 2738-117-VM | 10.06.2020 | 129 | NP | 3 | 99.9 | Environmental | Basel City |
| 2738-118-VM | 10.06.2020 | 129 | NP | 3 | 99.9 | Environmental | Basel City |
| 300093-18 | 04.01.2018 | 2 | 20 | 3 | 99.9 | Patient | Bern |
| 300237-19 | 10.01.2019 | 1 | 27 | 15 | 99.3 | Patient | Bern |
| 300337-17 | 18.01.2017 | 1 | NP | 32 | 98.6 | Patient | Basel City |
| 300337-20 | 18.01.2020 | 44 | NP | 6 | 99.7 | Patient | Basel City |
| 300382-20 | 20.01.2020 | 2 | NP | 4 | 99.8 | Patient | Basel City |
| 300442-18 | 20.01.2018 | unknown | Untypeable | 8 | 99.6 | Patient | Bern |
| 300482-17 | 26.01.2017 | 11 | 126 | 18 | 99.2 | Patient | Basel City |
| 300606-18 | 30.01.2018 | 11 | 45 | 36 | 98.4 | Patient | Bern |
| 300608-18 | 30.01.2018 | 5 | 23 | 25 | 98.9 | Patient | Basel City |
| 300664-17 | 09.02.2017 | 5 | 122 | 29 | 98.7 | Patient | Basel City |
| 300714-17 | 12.02.2017 | 130 | Untypeable | 13 | 99.4 | Patient | Basel City |
| 300720-20 | 06.02.2020 | 5 | NP | 30 | 98.7 | Patient | Basel City |
| 300732-16 | 09.02.2016 | 1 | NP | 64 | 97.2 | Patient | Bern |
| 300758-18 | 08.02.2018 | 11 | 78 | 20 | 99.1 | Patient | Basel City |
| 300775-18 | 08.02.2018 | 5 | 23 | 27 | 98.8 | Patient | Basel City |
| 300789-17 | 17.02.2017 | 11 | 78 | 18 | 99.2 | Patient | Basel City |
| 300791-18 | 09.02.2018 | 11 | 78 | 18 | 99.2 | Patient | Basel City |
| 300808-20 | 11.02.2020 | 11 | 78 | 18 | 99.2 | Patient | Bern |
| 300853-20 | 13.02.2020 | 372 | NP | 10 | 99.6 | Patient | Basel City |
| 300857-20 | 11.02.2020 | 14 | 14 | 2 | 99.9 | Patient | Luzern |
| 300873-17 | 23.02.2017 | 1 | 27 | 9 | 99.6 | Patient | Bern |
| 300900-19 | 25.02.2019 | 49 | 14 | 5 | 99.8 | Patient | Basel City |
| 300924-19 | 26.02.2019 | 55 | 57 | 6 | 99.7 | Patient | Basel City |
| 300944-17 | 01.03.2017 | 2 | 14 | 4 | 99.8 | Patient | Bern |
| 300944-18 | 20.02.2018 | 11 | 78 | 18 | 99.2 | Patient | Basel City |
| 300949-17 | 01.03.2017 | 6 | 5 | 7 | 99.7 | Ring Trial |  |
| 300965-19 | 01.03.2019 | 11 | 78 | 18 | 99.2 | Patient | Luzern |
| 300970-16 | 01.03.2016 | 1 | NP | 31 | 98.6 | Patient | Bern |
| 301032-19 | 03.03.2019 | 14 | NP | 2 | 99.9 | Patient | Basel City |
| 301078-18 | 28.02.2018 | 15 | 10 | 5 | 99.8 | Ring Trial |  |
| 301079-18 | 28.02.2018 | 6 | 5 | 7 | 99.7 | Ring Trial |  |
| 301099-20 | 27.02.2020 | 11 | NP | 18 | 99.2 | Patient | Basel City |
| 301102-19 | 06.03.2019 | 44 | 15 | 6 | 99.7 | Ring Trial |  |
| 301103-19 | 06.03.2019 | 2 | 14 | 4 | 99.8 | Ring Trial |  |
| 301114-16 | 02.03.2016 | 1 | NP | 63 | 97.2 | Patient | Bern |
| 301126-18 | 02.03.2018 | 5 | 23 | 26 | 98.9 | Patient | Basel City |
| 301144-17 | 16.03.2017 | 41 | 16 | 8 | 99.6 | Patient | Basel City |
| 301161-17 | 17.03.2017 | 1 | 27 | 173 | 92.4 | Patient | Bern |
| 301220-16 | 14.03.2016 | 1 | NP | 30 | 98.7 | Patient | Bern |
| 301227-18 | 06.03.2018 | 44 | 15 | 7 | 99.7 | Patient | Bern |
| 301234-18 | 06.03.2018 | 201 | 267 | 29 | 98.7 | Patient | Basel City |
| 301266-19 | 15.03.2019 | 54 | 12 | 3 | 99.9 | Patient | Basel City |
| 301297-18 | 09.03.2018 | 11 | 78 | 18 | 99.2 | Patient | Basel City |
| 301390-18 | 15.03.2018 | 6 | 5 | 6 | 99.7 | Patient | Basel City |
| 301391-18 | 15.03.2018 | 6 | 5 | 5 | 99.8 | Patient | Basel City |
| 301402-17 | 03.04.2017 | 11 | 126 | 19 | 99.2 | Patient | Basel City |
| 301413-18 | 18.03.2018 | 5 | 23 | 28 | 98.8 | Patient | Basel City |
| 301436-15 | 26.03.2015 | unknown | NP | 157 | 93.1 | Patient | Basel City |
| 301596-17 | 18.04.2017 | 5 | 23 | 27 | 98.8 | Patient | Basel City |
| 301608-20 | 28.03.2020 | 11 | 78 | 18 | 99.2 | Patient | Basel City |
| 301655-20 | 31.03.2020 | 11 | 126 | 19 | 99.2 | Patient | Basel City |
| 301699-18 | 04.04.2018 | 11 | 78 | 18 | 99.2 | Patient | Basel City |
| 301718-17 | 26.04.2017 | 47 | Untypeable | 5 | 99.8 | Patient | Basel City |
| 301729-17 | 27.04.2017 | 11 | 78 | 19 | 99.2 | Patient | Basel City |
| 301764-19 | 06.04.2019 | 16 | 50 | 11 | 99.5 | Patient | Luzern |
| 301861-20 | 15.04.2020 | 11 | 78 | 19 | 99.2 | Patient | Basel City |
| 301873-20 | 16.04.2020 | 11 | 126 | 19 | 99.2 | Patient | Basel City |
| 301875-17 | 06.05.2017 | 11 | 78 | 18 | 99.2 | Patient | Basel City |
| 302006-20 | 25.04.2020 | 8 | NP | 34 | 98.5 | Patient | Basel City |
| 302051-19 | 20.04.2019 | 201 | 267 | 29 | 98.7 | Patient | Basel City |
| 302078-18 | 23.04.2018 | 41 | Untypeable | 13 | 99.4 | Patient | Bern |
| 302094-18 | 23.04.2018 | 49 | 14 | 2 | 99.9 | Patient | Bern |
| 302112-17 | 22.05.2017 | 1 | 27 | 72 | 96.8 | Patient | Basel City |
| 302158-19 | 26.04.2019 | unknown | NP | 5 | 99.8 | Patient | Basel City |
| 302200-Mo1-19 | 29.04.2019 | 19 | 126 | 20 | 99.1 | Patient | Basel City |
| 302200-weissMo2-19 | 29.04.2019 | 11 | 126 | 18 | 99.2 | Patient | Basel City |
| 302205-weissMo1-19 | 29.04.2019 | 11 | 78 | 18 | 99.2 | Patient | Basel City |
| 302205-weissMo2-19 | 29.04.2019 | 286 | 78 | 5 | 99.8 | Patient | Basel City |
| 302221-17 | 30.05.2017 | 1 | 27 | 8 | 99.6 | Patient | Basel City |
| 302254-20-255 | 11.05.2020 | 8 | NP | 5 | 99.8 | Patient | Basel City |
| 302288-20 | 15.05.2020 | 8 | NP | 5 | 99.8 | Patient | Basel City |
| 302302-20 | 17.05.2020 | 237 | NP | 8 | 99.6 | Patient | Basel City |
| 302320-20 | 19.05.2020 | 11 | 126 | 20 | 99.1 | Patient | Basel City |
| 302339-19 | 06.05.2019 | 11 | 78 | 19 | 99.2 | Patient | Basel City |
| 302339-20 | 19.05.2020 | 11 | NP | 21 | 99.1 | Patient | Basel City |
| 302356-20 | 20.05.2020 | 11 | 78 | 21 | 99.1 | Patient | Basel City |
| 302357-20 | 20.05.2020 | 3 | NP | 6 | 99.7 | Patient | Basel City |
| 302358-20 | 20.05.2020 | unknown | NP | 22 | 99.0 | Patient | Basel City |
| 302386-20 | 22.05.2020 | 8 | NP | 5 | 99.8 | Patient | Basel City |
| 302409-17 | 09.06.2017 | 3 | 1 | 6 | 99.7 | Ring Trial |  |
| 302410-17 | 09.06.2017 | 8 | 2 | 4 | 99.8 | Ring Trial |  |
| 302422-19 | 11.05.2019 | 41 | 36 | 22 | 99.0 | Patient | Bern |
| 302444-18 | 15.05.2018 | 1 | 27 | 7 | 99.7 | Patient | Bern |
| 302451-17 | 13.06.2017 | 11 | 126 | 18 | 99.2 | Patient | Basel City |
| 302466-18 | 16.05.2018 | 5 | 23 | 28 | 98.8 | Patient | Basel City |
| 302488-18 | 17.05.2018 | 11 | 126 | 19 | 99.2 | Patient | Basel City |
| 302561-18 | 22.05.2018 | 55 | 70 | 6 | 99.7 | Patient | Basel City |
| 302568-18 | 22.05.2018 | 55 | 106 | 6 | 99.7 | Patient | Basel City |
| 302569-18 | 22.05.2018 | 55 | 70 | 6 | 99.7 | Patient | Basel City |
| 302572-20 | 04.06.2020 | 3 | NP | 9 | 99.6 | Patient | Basel City |
| 302652-20 | 11.06.2020 | 11 | NP | 18 | 99.2 | Patient | Basel City |
| 302796-18 | 06.06.2018 | 26 | 39 | 16 | 99.3 | Ring Trial |  |
| 302818-18 | 07.06.2018 | 11 | 78 | 18 | 99.2 | Patient | Solothurn |
| 302862-17 | 29.06.2017 | 11 | 126 | 18 | 99.2 | Patient | Basel City |
| 302880-19 | 05.06.2019 | 46 | 87 | 5 | 99.8 | Ring Trial |  |
| 302884-20 | 26.06.2020 | 3 | NP | 9 | 99.6 | Patient | Basel City |
| 302898-18 | 12.06.2018 | 55 | 70 | 161 | 92.9 | Patient | Basel City |
| 302898-18-2 | 12.06.2018 | 42 | 70 | 5 | 99.8 | Patient | Basel City |
| 302912-19 | 06.06.2019 | 55 | NP | 5 | 99.8 | Patient | Basel City |
| 302978-19 | 09.06.2019 | 55 | NP | 5 | 99.8 | Patient | Basel City |
| 303000-18 | 19.06.2018 | 55 | 70 | 7 | 99.7 | Patient | Basel City |
| 303068-20 | 07.07.2020 | 11 | NP | 21 | 99.1 | Patient | Basel City |
| 303136-18 | 29.06.2018 | 11 | 78 | 18 | 99.2 | Patient | Basel City |
| 303152-18 | 01.07.2018 | 5 | 23 | 27 | 98.8 | Patient | Basel City |
| 303187-18 | 02.07.2018 | 16 | 29 | 10 | 99.6 | Patient | Basel City |
| 303189-18 | 02.07.2018 | 18 | 50 | 7 | 99.7 | Patient | Basel City |
| 303194-20 | not provided | 8 | NP | 5 | 99.8 | Patient | Basel City |
| 303256-17 | 13.07.2017 | 1 | 27 | 7 | 99.7 | Patient | Basel City |
| 303270-17 | 13.07.2017 | 1 | 27 | 101 | 95.6 | Patient | Bern |
| 303331-18 | 10.07.2018 | 8 | 2 | 4 | 99.8 | Patient | Bern |
| 303378-18 | 13.07.2018 | 10 | 15 | 5 | 99.8 | Patient | Bern |
| 303392-18 | 14.07.2018 | 176 | 111 | 18 | 99.2 | Patient | Basel City |
| 303462-20 | 25.07.2020 | 5 | 23 | 27 | 98.8 | Patient | Basel City |
| 303481-18 | 19.07.2018 | 156 | 250 | 7 | 99.7 | Patient | Basel City |
| 303519-20 | 27.07.2020 | 8 | NP | 5 | 99.8 | Patient | Basel City |
| 303542-17 | 26.07.2017 | 11 | 126 | 19 | 99.2 | Patient | Basel City |
| 303584-7-20 | 30.07.2020 | 11 | 78 | 18 | 99.2 | Patient | Basel City |
| 303723-20 | 05.08.2020 | 11 | 126 | 18 | 99.2 | Patient | Basel City |
| 303759-17 | 06.08.2017 | 1 | 27 | 61 | 97.3 | Patient | Basel City |
| 303789-18 | 31.07.2018 | 1 | 27 | 8 | 99.6 | Patient | Bern |
| 303908-19 | 26.06.2019 | 11 | 33 | 36 | 98.4 | Patient | Basel City |
| 303958-17 | 15.08.2017 | 1 | 27 | 9 | 99.6 | Patient | Bern |
| 303999-17 | 17.08.2017 | 1 | 27 | 90 | 96.0 | Patient | Basel City |
| 304095-18 | 07.08.2018 | 54 | 12 | 2 | 99.9 | Patient | Basel City |
| 304107-20 | 26.08.2020 | 55 | 57 | 6 | 99.7 | Patient | Basel City |
| 304151-19 | 04.07.2019 | 5 | 23 | 27 | 98.8 | Patient | Basel City |
| 304173-20 | 30.08.2020 | 14 | NP | 2 | 99.9 | Patient | Basel City |
| 304178-20 | 30.08.2020 | 114 | 111 | 18 | 99.2 | Patient | Basel City |
| 304184-20 | 31.08.2020 | 98 | NP | 5 | 99.8 | Patient | Basel City |
| 304190-19 | 06.07.2019 | 11 | 78 | 18 | 99.2 | Patient | Bern |
| 304192-20 | 31.08.2020 | 14 | NP | 2 | 99.9 | Patient | Basel City |
| 304231-19 | 08.07.2019 | 6 | NP | 51 | 97.8 | Patient | Basel City |
| 304267-18 | 14.08.2018 | 99 | 70 | 6 | 99.7 | Patient | Basel City |
| 304275-18 | 14.08.2018 | 8 | 2 | 4 | 99.8 | Patient | Basel City |
| 304301-19 | 10.07.2019 | 21 | NP | 1 | 100.0 | Patient | Bern |
| 304353-19 | 11.07.2019 | 49 | NP | 2 | 99.9 | Patient | Basel City |
| 304388-20 | 11.09.2020 | 11 | 126 | 19 | 99.2 | Patient | Basel City |
| 304418-17 | 13.09.2017 | 2 | NP | 4 | 99.8 | Patient | Basel City |
| 304453-17 | 14.09.2017 | 11 | 78 | 18 | 99.2 | Patient | Basel City |
| 304465-17 | 15.09.2017 | 2 | NP | 4 | 99.8 | Patient | Basel City |
| 304518-20 | 18.09.2020 | 153 | NP | 10 | 99.6 | Patient | Basel City |
| 304589-19 | 31.07.2019 | 5 | 23 | 28 | 98.8 | Patient | Basel City |
| 304598-17 | 26.09.2017 | 11 | 78 | 20 | 99.1 | Patient | Basel City |
| 304625-17 | 27.09.2017 | 5 | 23 | 27 | 98.8 | Patient | Basel City |
| 304631-18 | 29.08.2018 | 6 | 5 | 6 | 99.7 | Ring Trial |  |
| 304649-17 | 26.09.2017 | 110 | NP | 5 | 99.8 | Patient | Basel City |
| 304800-18 | 31.08.2018 | 11 | 126 | 19 | 99.2 | Patient | Basel City |
| 304844-19 | 27.07.2019 | 67 | 153 | 11 | 99.5 | Patient | Basel City |
| 304848-19 | 27.07.2019 | 223 | 34 | 8 | 99.6 | Patient | Bern |
| 304907-17 | 10.10.2017 | 1 | 27 | 7 | 99.7 | Patient | Basel City |
| 304930-17 | 11.10.2017 | 42 | 106 | 4 | 99.8 | Patient | Basel City |
| 304936-Mo1-17 | 10.10.2017 | 11 | 126 | 18 | 99.2 | Patient | Basel City |
| 305091-20 | 24.10.2020 | 8 | NP | 4 | 99.8 | Patient | Basel City |
| 305095-19 | 06.08.2019 | 116 | NP | 10 | 99.6 | Patient | Basel City |
| 305107-20 | 26.10.2020 | 8 | NP | 4 | 99.8 | Patient | Basel City |
| 305128-17 | 24.10.2017 | 13 | 20 | 4 | 99.8 | Ring Trial |  |
| 305175-19 | 08.08.2019 | 122 | 131 | 17 | 99.3 | Patient | Basel City |
| 305284-19 | 02.08.2019 | 185 | 163 | 2 | 99.9 | Patient | St Gallen |
| 305299-16 | 17.12.2016 | 2 | 207 | 171 | 92.5 | Patient | Basel City |
| 305336-16 | 20.12.2016 | 2 | 207 | 10 | 99.6 | Patient | Basel City |
| 305393-16 | 24.12.2016 | 11 | 78 | 18 | 99.2 | Patient | Basel City |
| 305396-17 | 09.11.2017 | 45 | 13 | 11 | 99.5 | Patient | Bern |
| 305420-16 | 26.12.2016 | 22 | 23 | 26 | 98.9 | Patient | Basel City |
| 305469-17 | 14.11.2017 | 5 | 23 | 26 | 98.9 | Patient | Basel City |
| 305512-17 | 17.11.2017 | 5 | 122 | 30 | 98.7 | Patient | Bern |
| 305552-17 | 21.11.2017 | 42 | 106 | 4 | 99.8 | Ring Trial |  |
| 305553-17 | 21.11.2017 | 3 | 9 | 4 | 99.8 | Ring Trial |  |
| 305712-19 | 28.08.2019 | 10 | 15 | 4 | 99.8 | Ring Trial |  |
| 305739-18 | 09.10.2018 | 11 | 33 | 52 | 97.7 | Patient | Aarau |
| 305742-Mo1-17 | 04.12.2017 | 205 | Untypeable | 7 | 99.7 | Patient | Basel City |
| 305742-Mo2-17 | 04.12.2017 | 11 | 126 | 19 | 99.2 | Patient | Basel City |
| 305937-17 | 15.12.2017 | 92 | 150 | 4 | 99.8 | Patient | Bern |
| 306019-18 | 19.10.2018 | 41 | 251 | 9 | 99.6 | Patient | Basel City |
| 306159-19 | 15.09.2019 | 10 | 15 | 4 | 99.8 | Patient | Basel City |
| 306215-19 | 18.09.2019 | 11 | 78 | 18 | 99.2 | Patient | Bern |
| 306333-18 | 31.10.2018 | 11 | 78 | 18 | 99.2 | Patient | Bern |
| 306334-18 | 01.11.2018 | 41 | 251 | 9 | 99.6 | Patient | Bern |
| 306455-18 | 06.11.2018 | 54 | NP | 2 | 99.9 | Patient | Basel City |
| 306515-19 | 02.10.2019 | 11 | 78 | 19 | 99.2 | Patient | Basel City |
| 306530-18 | 09.11.2018 | 11 | 78 | 18 | 99.2 | Patient | Basel City |
| 306577-19 | 04.10.2019 | 11 | 78 | 19 | 99.2 | Patient | Basel City |
| 306808-18 | 20.11.2018 | 11 | 78 | 72 | 96.8 | Patient | Basel City |
| 306848-18 | 21.11.2018 | 2 | 20 | 5 | 99.8 | Ring Trial |  |
| 306907-18 | 22.11.2018 | 41 | Untypeable | 12 | 99.5 | Patient | Bern |
| 307063-19 | 01.11.2019 | 1 | 27 | 5 | 99.8 | Patient | Bern |
| 307281-19 | 10.11.2019 | 2 | NP | 4 | 99.8 | Patient | Basel City |
| 307354-19 | 16.11.2019 | 431 | NP | 15 | 99.3 | Patient | Bern |
| 307370-19 | 16.11.2019 | 393 | NP | 37 | 98.4 | Patient | Basel City |
| 307417-19 | 19.11.2019 | 6 | 5 | 7 | 99.7 | Ring Trial |  |
| 307418-19 | 19.11.2019 | 2 | NP | 5 | 99.8 | Ring Trial |  |
| 307614-19 | 28.11.2019 | 1 | 27 | 9 | 99.6 | Patient | Bern |
| 307728-19 | 05.12.2019 | 3 | NP | 6 | 99.7 | Patient | Basel City |
| 307793-18 | 27.12.2018 | 2 | 207 | 10 | 99.6 | Patient | Basel City |
| 307794-18 | 27.12.2018 | 2 | 207 | 10 | 99.6 | Patient | Basel City |
| 307989-19 | 16.12.2019 | 11 | 33 | 53 | 97.7 | Patient | Bern |
| 351237-20 | 17.01.2020 | 92 | NP | 7 | 99.7 | Patient | Basel City |
| 356841-18 | 14.04.2018 | 1 | 27 | 9 | 99.6 | Patient | Bern |
| 358658-20 | 27.04.2020 | 8 | NP | 5 | 99.8 | Patient | Basel City |
| 358659-20 | 27.04.2020 | 8 | NP | 5 | 99.8 | Patient | Basel City |
| 359591-19 | 29.05.2019 | 11 | 78 | 18 | 99.2 | Patient | Bern |
| 360013-20 | 02.06.2020 | 3 | NP | 6 | 99.7 | Patient | Basel City |
| 360432-18 | 31.07.2018 | 2 | 20 | 5 | 99.8 | Patient | Luzern |
| 360433-18 | 31.07.2018 | 2 | 20 | 10 | 99.6 | Patient | Luzern |
| 361438-20 | 08.07.2020 | 11 | NP | 18 | 99.2 | Patient | Basel City |
| 361897-18 | 04.09.2018 | 11 | 78 | 20 | 99.1 | Patient | Aarau |
| 363457-20 | 28.08.2020 | 13 | NP | 2 | 99.9 | Patient | Basel City |
| 363513-20 | 30.08.2020 | unknown | NP | 2 | 99.9 | Patient | Basel City |
| 363539-18 | 25.10.2018 | 4 | Untypeable | 7 | 99.7 | Patient | Bern |
| 363861-20 | 09.09.2020 | 5 | NP | 29 | 98.7 | Patient | Bern |
| 364710-20 | 29.10.2020 | 11 | 78 | 18 | 99.2 | Patient | Basel City |
| 368536-19 | 06.12.2019 | 11 | NP | 23 | 99.0 | Patient | Basel City |
| 368903-19 | 12.12.2019 | 102 | NP | 1 | 100.0 | Patient | Basel City |
| 62V | 25.02.2020 | 16 | NP | 9 | 99.6 | Environmental | Basel City |
| 63V | 25.02.2020 | 16 | NP | 9 | 99.6 | Environmental | Basel City |
| LU4505627 | not provided | 2 | 20 | 2 | 99.9 | Patient | Luzern |
| PRJEB37809: CdBe02 | 24.12.2016 | 1 | 27 | 25 | 98.9 | Patient | Bern |
| PRJEB37809: CdBe03 | 28.12.2016 | 1 | 27 | 35 | 98.5 | Patient | Bern |
| PRJEB37809: CdBe04 | 28.12.2016 | 1 | 27 | 70 | 96.9 | Patient | Bern |
| PRJEB37809: CdBe05 | 29.12.2016 | 1 | 27 | 13 | 99.4 | Patient | Bern |
| PRJEB37809: CdBe06 | 04.01.2017 | 1 | 27 | 18 | 99.2 | Patient | Bern |
| PRJEB37809: CdBe07 | 11.01.2017 | 1 | 27 | 41 | 98.2 | Patient | Bern |
| PRJEB37809: CdBe08 | 08.02.2017 | 1 | 27 | 145 | 93.6 | Patient | Bern |
| PRJEB37809: CdBe11 | 22.02.2017 | 1 | 27 | 217 | 90.4 | Patient | Bern |
| PRJEB37809: CdBe12 | 28.02.2017 | 1 | 27 | 200 | 91.2 | Patient | Bern |
| PRJEB37809: CdBe13 | 28.02.2017 | 1 | 27 | 211 | 90.7 | Patient | Bern |
| PRJEB37809: CdBe15 | 21.03.2017 | 1 | 27 | 62 | 97.3 | Patient | Bern |
| PRJEB37809: CdBe16 | 24.03.2017 | 1 | 27 | 105 | 95.4 | Patient | Bern |
| PRJEB37809: CdBe17 | 23.03.2017 | 1 | 27 | 172 | 92.4 | Patient | Bern |
| PRJEB37809: CdBe18 | 16.08.2017 | 1 | 27 | 32 | 98.6 | Patient | Bern |
| PRJEB37809: CdBe19 | 09.11.2017 | 1 | 27 | 92 | 95.9 | Patient | Bern |
| PRJEB37809: CdBe21 | 19.04.2017 | unknown | 27 | 107 | 95.3 | Patient | Bern |
| PRJEB37809: CdBe22 | 19.05.2017 | 1 | 27 | 21 | 99.1 | Patient | Bern |
| PRJEB37809: CdBe25 | 15.05.2018 | 1 | 27 | 130 | 94.3 | Patient | Bern |
| PRJEB37809: CdBe26 | 10.08.2018 | 1 | 27 | 31 | 98.6 | Patient | Bern |
| PRJEB37809: CdRi01 | 06.04.2017 | 1 | 27 | 45 | 98.0 | Patient | Bern |
| PRJEB37809: CdRi03 | 30.01.2018 | 1 | 27 | 117 | 94.8 | Patient | Bern |
| PRJEB37809: CdRi04 | 26.02.2018 | 1 | 27 | 9 | 99.6 | Patient | Bern |
| PRJEB37809: CdRi06 | 31.03.2018 | 1 | 27 | 52 | 97.7 | Patient | Bern |
| PRJEB37809: CdRi07 | 04.04.2018 | 1 | 27 | 14 | 99.4 | Patient | Bern |
| PRJEB37809: CdRi08 | 04.04.2018 | 1 | 27 | 29 | 98.7 | Patient | Bern |
| PRJEB37809: CdRi09 | 07.04.2018 | 1 | 27 | 74 | 96.7 | Patient | Bern |
| PRJEB37809: CdRi10 | 12.06.2018 | 1 | 27 | 8 | 99.6 | Patient | Bern |
| PRJEB37809: CdRi12 | 13.09.2018 | 1 | 27 | 9 | 99.6 | Patient | Bern |
| PRJEB43517: 103M | 02.06.2020 | 3 | NP | 9 | 99.6 | Environmental | Basel City |
| PRJEB43517: 109A | 03.06.2020 | 3 | NP | 9 | 99.6 | Environmental | Basel City |
| PRJEB43517: 109M | 03.06.2020 | 3 | NP | 9 | 99.6 | Environmental | Basel City |
| PRJEB43517: 111M | 10.06.2020 | 237 | NP | 8 | 99.6 | Environmental | Basel City |
| PRJEB43517: 117M | 10.06.2020 | 129 | NP | 3 | 99.9 | Environmental | Basel City |
| PRJEB43517: 118M | 10.06.2020 | 129 | NP | 3 | 99.9 | Environmental | Basel City |
| PRJEB43517: 128M | 29.06.2020 | 3 | NP | 9 | 99.6 | Environmental | Basel City |
| PRJEB43517: 131A | 30.06.2020 | 3 | NP | 9 | 99.6 | Environmental | Basel City |
| PRJEB43517: 131M | 30.06.2020 | 3 | NP | 9 | 99.6 | Environmental | Basel City |
| PRJEB43517: 21A | 04.12.2019 | 8 | NP | 5 | 99.8 | Environmental | Basel City |
| PRJEB43517: 41A | 21.01.2020 | 2 | NP | 6 | 99.7 | Environmental | Basel City |
| PRJEB43517: 41M | 21.01.2020 | 2 | NP | 6 | 99.7 | Environmental | Basel City |
| PRJEB43517: 43M | 22.01.2020 | 2 | NP | 6 | 99.7 | Environmental | Basel City |
| PRJEB43517: 44A | not provided | 11 | NP | 19 | 99.2 | Environmental | Basel City |
| PRJEB43517: 44M | not provided | 15 | NP | 5 | 99.8 | Environmental | Basel City |
| PRJEB43517: 45A | not provided | 5 | NP | 27 | 98.8 | Environmental | Basel City |
| PRJEB43517: 47A | not provided | 15 | NP | 5 | 99.8 | Environmental | Basel City |
| PRJEB43517: 47M | not provided | 15 | NP | 5 | 99.8 | Environmental | Basel City |
| PRJEB43517: 62A | not provided | 16 | NP | 9 | 99.6 | Environmental | Basel City |
| PRJEB43517: 62M | not provided | 3 | NP | 6 | 99.7 | Environmental | Basel City |
| PRJEB43517: 63A | not provided | 16 | NP | 9 | 99.6 | Environmental | Basel City |
| PRJEB43517: 63M | not provided | 3 | NP | 6 | 99.7 | Environmental | Basel City |
| PRJEB43517: 66A | 18.03.2020 | 11 | NP | 18 | 99.2 | Environmental | Basel City |
| PRJEB43517: 66M | 18.03.2020 | 11 | NP | 18 | 99.2 | Environmental | Basel City |
| PRJEB43517: 67A | 19.03.2020 | 11 | NP | 19 | 99.2 | Environmental | Basel City |
| PRJEB43517: 67M | 19.03.2020 | 11 | NP | 18 | 99.2 | Environmental | Basel City |
| PRJEB43517: 70A | 24.03.2020 | 33 | NP | 6 | 99.7 | Environmental | Basel City |
| PRJEB43517: 77M | 27.04.2020 | 54 | NP | 0 | 100.0 | Environmental | Basel City |
| PRJEB43517: 7A | 13.11.2019 | 2 | NP | 4 | 99.8 | Environmental | Basel City |
| PRJEB43517: 7M | 13.11.2019 | 2 | NP | 4 | 99.8 | Environmental | Basel City |
| PRJEB43517: 89M | 19.05.2020 | 2 | NP | 6 | 99.7 | Environmental | Basel City |
| PRJEB43517: 8A | 13.11.2019 | 2 | NP | 4 | 99.8 | Environmental | Basel City |
| PRJEB43517: 8M | 13.11.2019 | 2 | NP | 4 | 99.8 | Environmental | Basel City |
| PRJEB43517: 92M | 20.05.2020 | 11 | NP | 20 | 99.1 | Environmental | Basel City |
| PRJEB43517: 93M | 20.05.2020 | 93 | NP | 17 | 99.3 | Environmental | Basel City |
| PRJEB43517: Pt14 | 21.01.2020 | 110 | NP | 4 | 99.8 | Patient | Basel City |
| PRJEB43517: Pt16 | 22.01.2020 | 2 | NP | 6 | 99.7 | Patient | Basel City |
| PRJEB43517: Pt17 | 23.01.2020 | 5 | NP | 27 | 98.8 | Patient | Basel City |
| PRJEB43517: Pt19 | 18.01.2020 | 11 | NP | 19 | 99.2 | Patient | Basel City |
| PRJEB43517: Pt26 | 12.03.2020 | 11 | 78 | 19 | 99.2 | Patient | Basel City |
| PRJEB43517: Pt27 | 20.03.2020 | 33 | NP | 6 | 99.7 | Patient | Basel City |
| PRJEB43517: Pt30 | 04.03.2020 | 3 | NP | 6 | 99.7 | Patient | Basel City |
| PRJEB43517: Pt33 | 20.04.2020 | 54 | NP | 0 | 100.0 | Patient | Basel City |
| PRJEB43517: Pt43 | 19.05.2020 | 11 | NP | 20 | 99.1 | Patient | Basel City |
| PRJEB43517: Pt45 | 28.05.2020 | 5 | NP | 27 | 98.8 | Patient | Basel City |
| PRJEB43517: Pt47 | 05.06.2020 | 237 | NP | 8 | 99.6 | Patient | Basel City |
| PRJEB43517: Pt48 | 04.06.2020 | 129 | NP | 3 | 99.9 | Patient | Basel City |
| PRJEB43517: Pt9 | 03.12.2019 | 8 | NP | 5 | 99.8 | Patient | Basel City |

**Table S3. Comparison of ISR predictions from long read sequence assemblies, with band sizes from Bionumerics.** Grey numbers are the exported band sizes from Bionumerics, rounded to the nearest whole base. Due to the capillary sequencing method, closely sized bands are considered together. An offset of approximately 30bp is seen between the in silico and Bionumerics band sizes. Green rows show agreement between the methods and red cells are unexpected band sizes.

| Isolate | RT | Assembly | #*in silico* ISR amplicons | *In silico* ISR sizes | | | | | | | | | | | |
| --- | --- | --- | --- | --- | --- | --- | --- | --- | --- | --- | --- | --- | --- | --- | --- |
| 360432 | 020 | Bionumerics |  | 265/267 | 298 |  | 339/340 | 358/359 |  | 517/519 |  | 581 | plus 1 band > 600 |  |  |
|  |  | canu | 12 |  | 267 | 286/287 |  | 328/329 | 448 | 489 |  | 551/553 |  |  |  |
|  |  | canu_pilon | 12 | 234/236 | 267(2) |  | 309(2) | 329 |  | 488/489 | 533 | 551 |  |  |  |
|  |  | unicycler | 12 | 234 |  |  | 309 | 329 |  | 488 |  | 551 |  |  |  |
|  |  | flye | 12 |  | 267 | 286/287 |  | 329/330 |  | 488/489 |  | 551 |  |  |  |
|  |  | flye_pilon | 12 | 234 | 267 |  | 309 | 329 |  | 488/491 |  | 551 |  |  |  |
| 302561 | 070 | Bionumerics |  |  |  |  |  | 340 |  | 357 | 398/400/402 | 415 | 517/519/520 | 581 |  |
|  |  | canu | 11 |  |  | 234/236 | 267 | 309 |  | 328 |  |  | 486/488 | 551 | 573 |
|  |  | canu_pilon | 11 | 225/227 |  |  |  | 309 |  | 328 |  | 384 | 488/489 | 551 | 575 |
|  |  | unicycler | 8 |  |  |  |  | 309 |  | 329 |  |  | 491 | 551 |  |
|  |  | flye | 12 |  |  | 234 | 267 | 309 |  | 327 |  |  | 490 | 550/551 |  |
|  |  | flye_pilon | 11 | 156 | 171 | 233/234 |  | 309 | 314 | 327 |  |  | 488/491 | 551 |  |
| 306515 | 078 | Bionumerics |  | 338 | 358/361 | 402/404 | 446 | 477 | 521 |  |  |  |  |  |  |
|  |  | canu | 18 | 309 | 329/331 | 370/373 | 415 | 447/448/449 | 490/491 |  |  |  |  |  |  |
|  |  | canu_pilon | 18 | 311 | 329/331 | 371/373 | 415 | 447/448/449 | 490/491 |  |  |  |  |  |  |
|  |  | unicycler | 12 | 311 | 329/331 | 371/373 | 415 | 449 | 491 |  |  |  |  |  |  |
|  |  | flye |  | 311 | 329/331 | 373 | 415 | 449 | 491 |  |  |  |  |  |  |
|  |  | flye_pilon | 12 | 311 | 329/331 | 373 | 415 | 449 | 491 |  |  |  |  |  |  |
| 359591 | 078 | Bionumerics |  |  |  |  | 338 | 358/361 |  | 402/404 |  | 446 | 477 | 521 |  |
|  |  | canu | 12 |  |  |  | 309 | 329 |  | 371/373 | 384 |  |  | 487/489/491 | 550 |
|  |  | canu_pilon | 12 | 241 | 262 |  | 311 | 331 | 346 | 369/373 | 384 |  |  | 491 | 550 |
|  |  | unicycler | 10 |  |  |  |  | 329/331 |  | 371/373 |  |  | 449 | 491 | 533 |
|  |  | flye | 12 |  |  |  | 309 | 329 |  | 371/373 | 384 |  | 449 | 489/491 | 551 |
|  |  | flye_pilon | 13 | 242 | 255 | 303 | 311 | 329/331 |  | 373 | 384 |  | 449 | 489/491 | 551 |
| 301392 | 126 | Bionumerics |  | 339 | 358/361 | 402/404 | 478 | 521 |  |  |  |  |  |  |  |
|  |  | canu | 12 | 311 | 329/331 | 371/373 | 448/449 | 491 |  |  |  |  |  |  |  |
|  |  | canu_pilon | 11 | 311 | 329/331 | 371/373 | 448/449 | 491 |  |  |  |  |  |  |  |
|  |  | unicycler | 12 | 311 | 329/331 | 371/373 | 449 | 491 |  |  |  |  |  |  |  |
|  |  | flye | 12 | 311 | 331/332 | 373 | 448/449 | 491 |  |  |  |  |  |  |  |
|  |  | flye_pilon | 11 | 311 | 329/331 | 371/373 | 448/449 | 491 |  |  |  |  |  |  |  |
| 302200 | 126 | Bionumerics |  | 339 | 358/361 | 402/404 |  | 478 | 521 |  |  |  |  |  |  |
|  |  | canu | 13 | 311 | 329/330 | 371/373 | 414/415 | 448/449 | 491 |  |  |  |  |  |  |
|  |  | canu_pilon | 13 | 311 | 329/331 | 371/373 |  | 448/449 | 491 |  |  |  |  |  |  |
|  |  | unicycler | 12 | 311 | 329/331 | 371/373 |  | 449 | 491 |  |  |  |  |  |  |
|  |  | flye | 12 | 312 | 331 | 373 | 415 | 449 | 491 |  |  |  |  |  |  |
|  |  | flye_pilon | 12 | 311 | 329/331 | 373 | 415 | 449 | 491 |  |  |  |  |  |  |
